# Unusual Cause of Gastrointestinal Bleeding in an Elderly Adult: Gastric Kissing Ulcers

**DOI:** 10.5152/tjg.2024.23647

**Published:** 2024-05-01

**Authors:** Osman Yüksekyayla, Ersin Batıbay, Cumali Efe

**Affiliations:** Department of Gastroenterology, Harran University, Şanlıurfa, Türkiye

## Dear Editor,

Upper gastrointestinal bleeding (UGIB) is a medical emergency and is associated with high mortality and morbidity. UGIB is defined as bleeding in the esophagus, stomach, or proximal part of duodenum.^1^ Peptic ulcers resulted from *Helicobacter pylori* infection and the use of nonsteroidal anti-inflammatory drugs (NSAIDs) or antithrombotic agents, are the most common cause of UGIB worldwide.^1,2^ Kissing ulcers are defined as the pair of ulcers facing each other on the opposite walls of the stomach or duodenum.^3^ Kissing ulcers are commonly observed in the duodenum but very rarely seen in the stomach. We report a case of kissing gastric ulcers presenting as melena in a patient on warfarin therapy.

**Figure 1.**
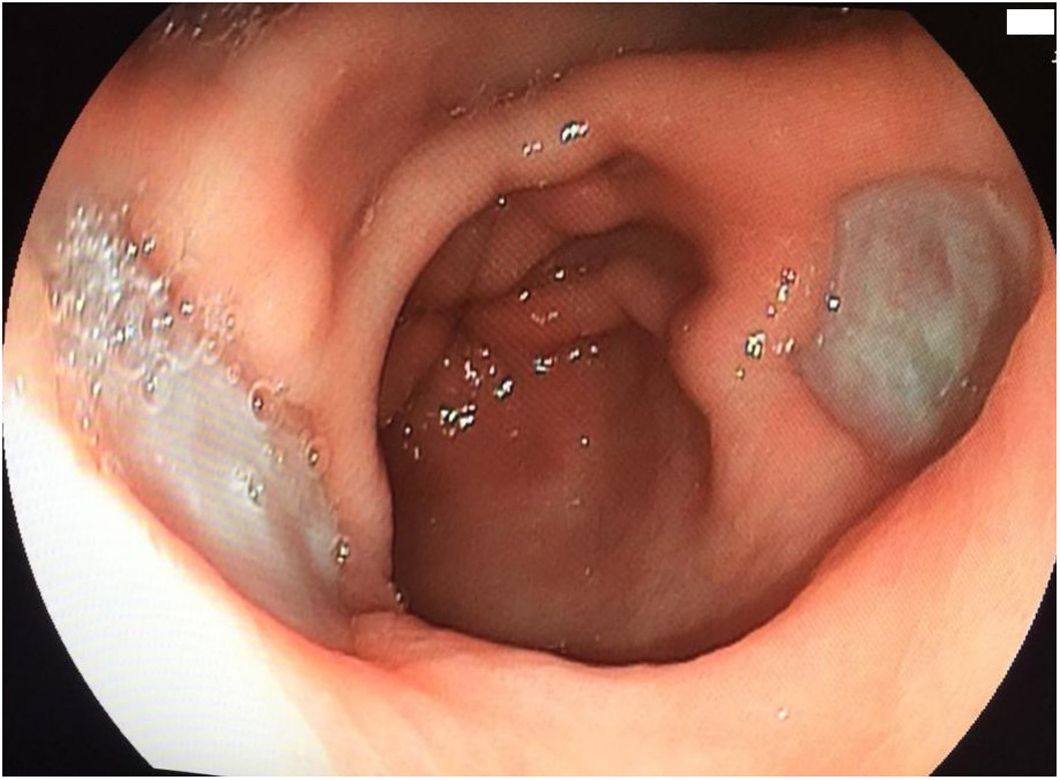
Kissing ulcers are seen in the anterior and posterior walls of the distal corpus.

A 82-year-old man was admitted to the hospital for abdominal pain and melena. The patient was under anticoagulation with warfarin (5 mg/daily) because of mitral valve replacement that performed 10 years earlier. On examination, blood pressure was 105/65 mm Hg and heart rate was 105 beats/min. Abnormal laboratory results were as follows: hemoglobin 8.4 g/dL, hematocrit 26, and international normalized ratio (INR) 2.4. Previous hemoglobin and INR levels were 12.2 g/dL and 2.6 respectively, approximately 6 weeks ago. An upper gastrointestinal endoscopy showed 2 circular, kissing ulcers which had clean bases covered by whitish exudate, and surrounded by normal gastric mucosa without active bleeding. They were located in the anterior (size, 27 × 20 mm) and posterior walls (size, 15 × 20 mm) of the distal corpus (Figure 1). Biopsy samples from the margin and base of the ulcers did not reveal any malignancy and helicobacter pylori was negative. He was treated with a proton pump inhibitor (rabeprazole 2 × 20 mg/day) and discharged from the hospital with no complications in a week.

Kissing gastric ulcers are very rare cause of gastrointestinal bleeding as we could identify only 6 such cases reported in the literature.^3-8^ In previous reports, development of gastric kissing ulcers was attributed to abdominal blunt trauma in 4 cases,^3,6-8^ NSAIDs intake in 1 case,^4^ and the etiology was unknown in another case.^5^ Our patient did not have any history of trauma or the use of NSAIDs. Only the use of warfarin might be a risk factor for the development of gastric ulcers.

A previous experimental study showed that proton pump inhibitors effectively induced mucosal healing of kissing gastric ulcers in rats.^9^ Similar to previous reports,^3,6,7^ we also successfully treated our patient with proton pump inhibitors. The outcome of reported cases with kissing gastric ulcers was generally good, except in 1 case, kissing gastric ulcers complicated by acute pancreatitis and portal biliopathy.^5^ This adverse outcome suggests that early diagnosis and prompt treatment of kissing gastric ulcers are crucial.

We described a case of kissing gastric ulcer, which was diagnosed in a patient on warfarin therapy. Though rare, gastric kissing ulcers should be considered among the differential diagnosis of patients presenting with upper gastrointestinal bleeding.
